# Healthy and sustainable diets for future generations

**DOI:** 10.1002/jsfa.8953

**Published:** 2018-03-25

**Authors:** Hilary Green, Pierre Broun, Douglas Cook, Karen Cooper, Adam Drewnowski, Duncan Pollard, Gary Sweeney, Anne Roulin

**Affiliations:** ^1^ Nutrition, Health and Wellness Unit Nestlé Research Centre Lausanne Switzerland; ^2^ Nestlé Research and Development Centre Tours France; ^3^ Department of Plant Pathology University of California Davis USA; ^4^ Center for Public Health Nutrition University of Washington Seattle USA; ^5^ Stakeholders Engagement in Sustainability, Operations, Nestec SA Vevey Switzerland

**Keywords:** nutrition, agriculture, sustainability, food security, plant science

## Abstract

Global food systems will face unprecedented challenges in the coming years. They will need to meet the nutritional needs of a growing population and feed an expanding demand for proteins. This is against a backdrop of increasing environmental challenges (water resources, climate change, soil health) and the need to improve farming livelihoods. Collaborative efforts by a variety of stakeholders are needed to ensure that future generations have access to healthy and sustainable diets. Food will play an increasingly important role in the global discourse on health. These topics were explored during Nestlé's second international conference on ‘Planting Seeds for the Future of Food: The Agriculture, Nutrition and Sustainability Nexus’, which took place in July 2017. This article discusses some of the key issues from the perspective of three major stakeholder groups, namely farming/agriculture, the food industry and consumers. © 2018 The Authors. Journal of The Science of Food and Agriculture published by John Wiley & Sons Ltd on behalf of Society of Chemical Industry.

## INTRODUCTION

The global discourse around food systems has historically been on production issues. Actions have largely focused upon reducing the environmental impact of our existing systems. This is, however, changing. We can now grow enough food for today's needs and even those for the world's population in 2050. The challenge now is one of health. We face unprecedented human health challenges that our food system can and must deliver. It is time to shift the focus from farm to fork, and now think of fork to farm. In other words, the focus should now be ‘healthy foods from healthy soils’.

Defining what is meant by a “sustainable diet” is both broad and complex. It encompasses the entire food supply chain, and takes account of health, the environment, affordability and culture.^1^ Future food systems will need to provide a rapidly growing population with foods that are not just both affordable and nutrient rich, but are also restorative in terms of their impact on land, water and energy resources. Such changes can come about through both shaping food supply and increasing the demand for healthy food.

Nestlé's second international conference on ‘Planting Seeds for the Future of Food: The Agriculture, Nutrition and Sustainability Nexus’ aimed to promote a better understanding of the respective and interacting roles of the global consumer and the food supply chain. Facilitating and promoting the consumption of healthy and sustainable diets was the principal theme. The conference, which took place in July 2017 (the programme is provided as supporting information), was the second in an ongoing series^2^ and served to highlight the need for a joint effort from a diverse range of stakeholders to address global food needs. The conference attracted 160 external participants from non‐government organizations, international organizations, academia and the private sector, as well as approximately 300 internal Nestlé participants and a further 250 who followed the webcast sessions.

The key theme of the event was ‘the nexus of agriculture, nutrition and sustainability’. The first day focused on agriculture, with an eye on nutrition and health outcomes, while the second day focused on nutrition and the consumer, but with linkages back to agriculture.

The ideas presented during the conference, and ensuing debates, were broadly relevant to a variety of different stakeholders, including farmers, the food industry and consumers.

## FARMING

In high‐income countries food is typically viewed today as a commodity where economic profit is a major objective rather than a source of nutrition and health benefits.^3^ It is estimated that modern farming systems produce sufficient calories to meet current demand, but this generalization belies the many factors, often local, that limit production, reduce choice and constrain access.^4^, ^5^ Thus identifying solutions that can improve agricultural production, while providing access to healthy, nutritious foods may be the best approach in addressing food security.^6^ The intensification of agriculture in the last century has been a key factor in meeting growing food demand. However, intensive agricultural practices are generally not sustainable, having degraded approximately 33% of the Earth's arable soil, increased greenhouse emissions, reduced pollinator populations, strained supplies of available fresh water, and endangered fresh and marine wildlife, resulting in a modern farming system in urgent need of change.^7^

Efficient knowledge transfer from agricultural research to farmers is a key step to advance sustainable farming practices, with the goal of improving the environmental impact of agriculture – in particular, restoring soil health, protecting ecosystems and biodiversity – while ensuring a stable, productive and quality supply of food.

The current food and agriculture system must change if we are to meet the UN's second sustainable development goal (SDG2) to end hunger and achieve food security and improved nutrition, while promoting sustainable agriculture. Agricultural practices must also address climate change, to which modern, energy‐intensive agriculture is a significant contributor. Although climate models differ in their specific predictions,^8^ it is generally agreed that near‐term sustained temperature increases will yield more extreme and variable weather, including extended droughts and flooding,^9^ which would adversely shift the dynamics of agriculture and disrupt global food supplies. Interestingly, meta‐analyses for a range of crop species have shown that future increases to atmospheric CO_2_ will also impact the nutritional protein content of crops^10^ as well as minerals such as zinc and iron.^10^, ^11^, ^12^ Hence, to maintain or improve an individual's nutritional profile, it will remain important to make available additional varieties of nutrient‐rich foods, such as meat, dairy, fish, nutrient‐rich vegetables/legumes and fortified products, but in a more feasible manner for the environment than that of the current food and agriculture system. Sustainable agricultural practices should also focus on the rehabilitation of degraded soils, which limit agricultural productivity and in some parts of the world is a driving force in the conversion of pristine environments to agriculture uses.

### Seeds

Seed quality and supply impact the volume and nutritional value of the crops.^13^ Seed systems in Africa are generally underdeveloped, with the majority of smallholder farmers procuring seed through informal systems, and therefore commercial seed companies making only minor contributions.^14^ The absence of effective seed systems reduces productivity because poor seed quality impacts seedling emergence, vigour and yield potential, and because modern crop varieties are often not locally available.^14^ The recently developed *Access to Seeds Index* 15 addresses this issue by providing performance indicators for how well the seed industry is addressing the needs of smallholder farmers. One challenge is that most of the research investment focuses on developing better plant varieties for a limited number of crops. This narrow focus fails to incorporate minor species and varieties that may be locally preferred and comprise the mainstay of local diets. Thus there is a need for increased private and public sector investment in crops that are regionally important, although perhaps minor on a global scale, especially those that are preferred by consumers and have a high nutritional value. Seed systems are also the distribution pathway for crop‐specific products, such as rhizobial inoculants and fungicides. Tailoring these inputs to meet specific agronomic requirements for Africa, which can be highly diverse, and to do so in a way that is both affordable to the farmer and sustainable for the environment, is a significant challenge.

### Plant science

Consideration must also be given to the choice of crops being grown. Over the last 50 years, agricultural homogenization has meant a decrease in crop diversity.^16^ Fifteen crops currently provide ∼90% of humankind's caloric intake, including three crops – wheat, rice and maize – that account for about two‐thirds of dietary energy.^17^ Of equal importance, the genetic diversity within individual crop species is low, owing to genetic bottlenecks during domestication and to the replacement of a wide range of landraces by a few highly productive varieties in major crop species. This reduction in diversity, combined with a historical focus of genetic selection on output, is limiting the farmer's options to grow crops that are both nutritionally dense and resilient to climate factors and disease. This situation, in turn, affects food sufficiency, as well as dietary breadth and quality.^16^, ^18^ These and related issues are of particular concern for communities that rely heavily on plant‐based diets.

Landraces and crop wild relatives represent our agricultural heritage, which provide the genetic ‘building blocks’ for plant improvement.^19^ Leveraging their resilience and adaptations in modern agriculture is an urgent undertaking, owing to the value they can bring to modern crops and because, in the absence of collection and conservation, they will just disappear. Classically, crop relatives were the source of individual traits, most commonly disease resistance,^20^ but with recent advances in genome‐scale approaches the stage is set for a more systematic characterization and use of crop wild relatives. Such an approach is being undertaken for chickpea to improve a range of agronomic traits, including tolerance to climatic extremes.^21^, ^22^, ^23^ Similar approaches are feasible to improve the nutritional content of crops, as has been done, for example, in corn for provitamin A. Modern plant breeding is leveraging knowledge of genome content and function, in order to precisely select for genes that underlie high‐value traits. In this context, genome editing by CRISPR/CAS9, which allows the specific targeting of genes, could be the next frontier in improving crops for nutritional value,^24^ provided the appropriate regulatory framework is put in place.

Exploration of new technologies should also be considered: for example, emerging devices to monitor crop growth and physiology in real time, enhanced soil quality through microbial amendments, and tracking disease risk to improve crop growth and livestock well‐being. However, will these technologies really enhance yield and nutritional quality? Or is it just more work for the farmers? The FAO estimated 20–40% of crop yields are lost each year to pests and disease, and technology identifying such problems early can improve productivity.^25^ Additionally, monitors of water irrigation systems can help conserve water^26^ and limit the systemic overuse of global freshwater resources – humans currently use ∼70% of available fresh water, most intensively for agricultural purposes.^27^ Recent research suggests that 66% of the human population live under severe water scarcity at least one month of the year.^28^ Introducing such practices will enable farmers to focus on producing nutritionally rich produce, while improving biodiversity, conserving water, restoring natural lands and reducing food waste in an attempt to move within planetary boundaries outlined by the Stockholm Resilience Centre.^29^

Arguably, one of the greatest obstacles for future farming systems may be to ensure that the benefits of science are understood and that scientific advances meet the expectations of consumers.^30^ Balanced engagement of public media and policymakers will be essential, moving beyond the traditional debate about the merits of specific technologies (e.g. genetically modified organisms (GMOS) and breeding practices such as CRISPR/CAS9) and focusing on the quality and desirability of outcomes.

### The farming workforce

Data from the World Bank estimate that the global agricultural labour force has been reduced in recent years from 34.8% in 2005 to 29.5% in 2010,^31^ mainly due to increases in agricultural mechanization and technologies. Nevertheless, attracted by urban employment and social opportunities, the children of farmers leave rural farmland areas.^32^ The UN World Health Organization predicts that six out of ten people will live in a city by 2030, resulting in fewer young people being willing to work on rural farms and the associated issues related to large‐scale migration. It is also likely that there will be fewer young people in agricultural institutes that are important influencers for the general view of agriculture and decision making in rural and agricultural affairs. Consequently, an appreciation and understanding of farming systems is being lost. Farmers, young and old, also need to reconnect with consumers to facilitate a better mutual understanding of the value of food and the challenges in agriculture.

Retaining young farmers has become a major problem in developing countries. One organization trying to resolve this is the International Institute of Tropical Agriculture (IITA).^33^ The IITA help engage and educate youth graduates in the area of agriculture as well as meet pressing challenges of relieving hunger, malnutrition, poverty and natural resource degradation. Innovative programs such as the IITA Youth Agripreneurs (IYA) and the ENABLE YOUTH programs help educate youths to engage and develop agriculture and business skills, adopting improved technology and value chain options with a holistic approach. One example of the IITA's success is the work to support vegetable production and market presence in Uganda. Here, the development of online food ordering applications (vegetable online basket),^34^ provides a market practice in which youths can engage through a system that is exciting and far from stereotypical of traditional farming.

Through providing youth grants, education, training and engagement from organizations like the IITA, the future farming workforce can grow and develop sustainable farming practices. However, an interest in farming needs to be continuously stimulated; thus funding and protection are needed from policymakers, not just for future farmers but for current farmers. Policies must align farmers with a clear commitment from the market to protect and ensure profits for sustainable farming systems. Protecting farmers' economic value can provide not only growth of global agriculture but also impact nutrition security for the farmer and the surrounding communities through multiple channels of employment, education and lifestyle.

## FOOD INDUSTRY

The food industry is incredibly diverse, from the companies that provide the seeds and the agricultural inputs to the diverse food companies that transform ingredients into products and the retailers and restaurants that put these products onto shelves and on our menus. The food value chain is highly fragmented, comprising start‐ups, small and medium enterprises right through to large multinational corporations. Therefore, it is imperative that all parts of the food industry recognize how their actions can, and do, influence health and environmental sustainability across the food system.

Industries that are consumer facing, i.e. branded product makers, supermarkets, restaurants and so on, have a unique opportunity to influence diets. In every interaction, there is the potential to nudge consumers towards a healthier and more sustainable choice.^35^, ^36^ The food environment today is rich with information and choice architecture that can inform or confuse a consumer. Industry can better use nudging, choice editing, pricing, innovation and general promotion to help consumers to make the best choice. Increasing the nutrient density of both fresh and packaged products would be one avenue that should be welcomed, though what would be the costs of such changes? Ideally, the best choice should be the easiest choice.

Food companies are subjected to fluctuations in raw material prices,^37^ while certain commodities are subject to subsidies that can distort the food system, with implications for human health.^38^, ^39^ Some organizations are promoting true costing of social and natural capital, which is the internalization of societal and environmental costs that make up a product. It remains to be seen if this approach is taken up systemically. However, alignment of policies towards nutrition, rather than just agriculture, to reconfigure the subsidy systems in place would seem a logical step.

Industry is impacting the next generation through what it delivers to consumers today. Childhood diets are often set early,^40^ meaning the early years of life should be seen as the period to shape healthy and sustainable diet preferences. Industry can help shape that with products that are congruent with this ideal. Nutrient profiling systems that provide a means to evaluation the nutritional value of products, including those made for children, are useful in product renovation and innovation^41^ as well as for restricting marketing to children.^42^ Partnering this with increased spending on research and development to create affordable, nutrient‐dense, responsible and profitable products that are welcomed into a healthy and sustainable dietary framework would be ideal.

Consumers, however, may not yet desire this approach, and so may not reward the food industry for making moves in this direction. Society should therefore step in, which implies that a systemic approach towards change would be needed rather than a linear approach to only deliver what consumers expect today. Maintaining consumer trust in the food industry while making these changes will be essential.

## CONSUMERS

### Protein–energy malnutrition and micronutrient deficiencies

Protein–energy malnutrition and micronutrient deficiencies continue to affect millions of people worldwide.^43^ For example, around half of the world's maternal and child population is affected by undernutrition and deficiencies in micronutrients, notably iron, iodine, vitamin A and zinc.^44^

One avenue to address micronutrient deficiencies is biofortification. This involves the breeding of plant varieties that are richer in a particular mineral or micronutrient. HarvestPlus, together with its partners, has been pioneering efforts to deliver biofortification on a global scale since 2003.^45^ They have created versions of key raw materials that are more nutrient dense than their counterparts that are cultivated today, namely rice, wheat, maize, pearl millet, sorghum, banana/plantain, potato, lentil, beans and cassava. If these became the mainstream versions of these key food crops, much better nutrition would filter affordably to millions of people. Scaling up the production of these varieties is the main challenge today, and the food producers could play a role here by stimulating the supply chain. If companies were to create more nutrient‐dense products, then it would be logical to specify minimum contents of key nutrients in their raw materials. Farmers could choose to grow these crops with less risk, and seed companies would be provided with a strong business case to transition to more nutrient‐dense seed supply. HarvestPlus has reached 4.2 million households so far, with a goal to reach 20 million by 2020. Whether this is possible without the food industry remains an open question.

### Nutrition transitions

With increasing economic development and urbanization, there are dietary changes or ‘nutrition transitions,’ characterized by being of poor nutritional quality and high in calories,^46^, ^47^, ^48^ but not widely affordable or sustainable.^46^, ^47^, ^49^

These nutrition transitions are creating a number of nutrition‐related problems, including new obesity hotspots as people switch from traditional diets to more western diets. Another important issue with diets that are undergoing this nutrition transition is the demand for animal protein, which is relatively expensive and, as is well documented, is generally less environmentally sustainable than plant protein.^50^

In many developed countries, meat is the dominant dietary component, even though this is not necessary from a nutritional point of view. Conversely, in much of the developing world, where meat would be a useful addition to the diet, it is neither available nor affordable. Individuals and families can adapt to food price shocks by reducing their consumption of animal proteins as well as by focusing dietary intake on staple foodstuffs. However, this may result in a decrease in the dietary diversity that is needed for good health.

As societies gain affluence, the percentage of income devoted to food continues to decrease, while other household expenses increase. However, access to food, including affordability, is still a grave and pressing concern for those at the bottom of the economic pyramid in both developing and developed countries. Hunger is also linked to social unrest, often in the very communities that grow important raw materials.^51^ Last year's *Global Nutrition Report*, as well as the HLPE's *Nutrition and Food Systems Report*, address the global nutrition situation and the ways that the Sustainable Development Goals provide an opportunity for change.^52^, ^53^

### Sustainable diets for communities and individuals

Population growth and urbanization are key drivers for changing the food system and food‐based dietary guidelines. A number of considerations are paramount for ensuring that healthy diets are also sustainable diets – not only their environmental and health impact but also price, taste, culture and convenience. The links between socio‐economic class and diet quality are becoming well documented in different cultures.^54^, ^55^, ^56^, ^57^

The ultimate goal for the food system has to be to help individuals and families enjoy a high quality of life by making available food and nutrition that delivers health needs. To some extent, this is already done when an individual has been diagnosed with a particular diet‐related problem. For example, special diets are needed for people with inborn errors of protein metabolism such as phenylketonuria (PKU),^58^ and dietary changes are recommended for people with dyslipidaemia, hypertension or impaired glucose tolerance.^59^ Increasingly, however, it will be possible to personalize dietary advice according to dietary preferences, biological data and activity levels. However, this is not just a question of getting biological information (genetic, metabolic etc.) about individuals – it is also about having the right communication and channels to drive behaviour change. Indeed, evidence from the Food4Me trial suggests that personalized nutrition advice, delivered for 6 months via the Internet, is more effective in changing dietary behaviour than conventional dietary advice.^60^ Consequently, companies that invest in digital technologies that provide personalized dietary recommendations will not only help individuals eat more healthily, but potentially also this will lead to societal benefits in terms of public health.

## CONCLUSIONS

The Nestlé conference tackled numerous complex topics that have implications for farmers, the food industry, individuals and families. Numerous questions were asked and long‐standing ideas were challenged. The need for more systems thinking pervaded the event, illustrated by two long‐standing ideas: the notion of linearity in the food system, and the notion of doubling food production by 2050.

The idea of linearity in the food system refers to the concept that food passes in a linear fashion from the farmer to the consumer (‘farm to fork’). Rather than being a linear process, the food system is actually a network or matrix. While systems thinking can help us, we should not get paralysed by the interconnectedness of everything. We need to pick a few things and go after them.

Such systems thinking led to a second insight: the conference challenged the conventional wisdom that we need to more than double food production by 2050. By tackling nutrition loss and waste, and raising more grass‐fed cattle through rotational farming, we can go a long way to nourishing the world and ensuring that soils are preserved to continually focus upon yields.

Such system thinking provided the hope that a collaborative effort by all the stakeholders could help to tackle some of the food challenges we are facing. It will be necessary to re‐evaluate our relationship with the planet by thinking about the wellness of both the planet and ourselves, and not to view the planet's health and human health as separate issues.

In the spirit of Sustainable Development Goal 17, new multi‐stakeholder partnerships that mobilize and share knowledge, expertise, technology and financial resources must be created to build on the experience and resourcing strategies of the various partners. This will mean breaking down silos of scientific and technical disciplines and also barriers between private and public institutions and civil society organizations to build the required collaborative effort.

## Supporting information


**Appendix S1.** Supporting information
