# Supplementary material for: Healthy and sustainable diets for future generations
Source: J Sci Food Agric. 2018 Mar 25;98(9):3219–24. doi: 10.1002/jsfa.8953 (PMC6033153; doi:10.1002/jsfa.8953)

Nestlé

# PLANTING SEEDS FOR THE FUTURE OF FOOD II

## THE AGRICULTURE, NUTRITION AND SUSTAINABILITY NEXUS

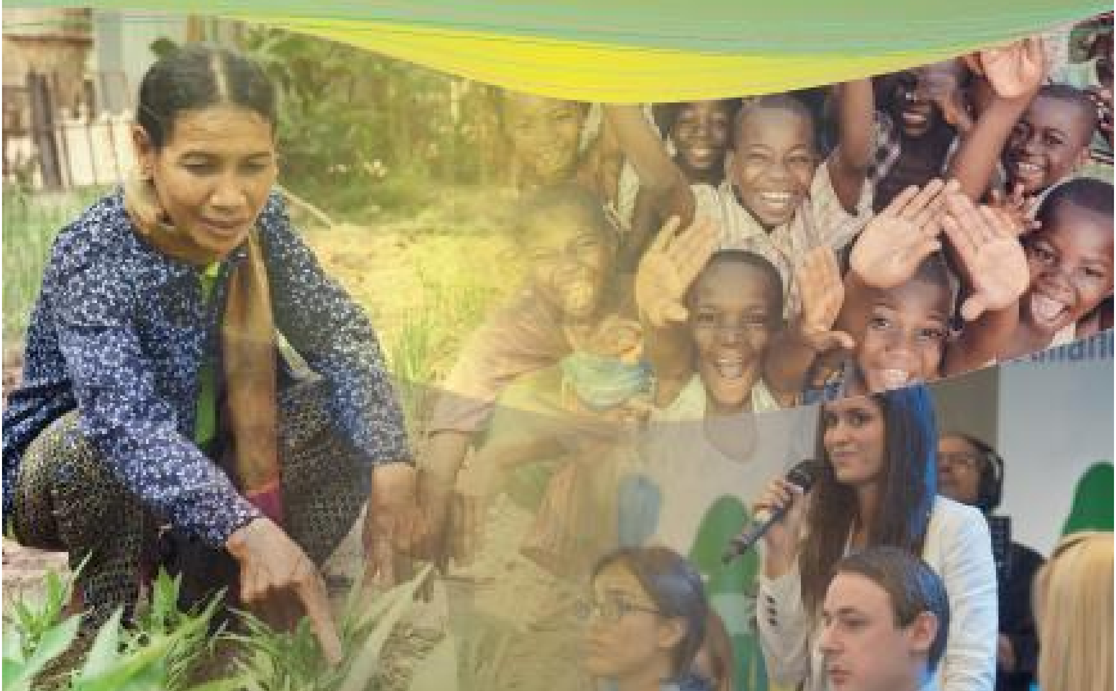

## COME AND JOIN THE DEBATE

### 2-day event of plenary sessions, panel debates and demos

Nestlé Headquarters Vevey, Switzerland
July 6–7, 2017

## Plenary sessions web casts

| Day 1: July 6 | Theme Day 1 Sustainable Farming Systems | |
|---|---|---|
| *08.30-09:00* | *Coffee, refreshments* | |
| 09:00-09:10 Link to the webcast | Welcome and opening remarks | Magdi Batato, *Head of Corporate Operations, Nestlé* |
| 09:10-10:30 Link to the webcast | **Plenary: Setting the scene**<br>What Has Nature Ever Done for Us?<br>Sustainable Food Systems<br><br>Productive Capacity of Farming Systems<br><br>Consumers, Big Food, Urbanisation | **Plenary Area**<br>Tony Juniper<br>Ruth Richardson *Global Alliance for the Future of Food*<br><br>Patrick Holden *Sustainable Food Trust*<br><br>Magdi Batato |
| 10:30-11:00 | *Refreshment break* | |
| 11:00-12:30 | **Deep Dive Session 1**<br>**Healthy Soils, Healthy Plants, Healthy Food**<br>Choice of 4 workshops | • Biodiversity for the planet and for food Bev Postma *HarvestPlus*, Stefano Padulosi *Bioversity,* Tony Juniper, Urs Schenker *Nestlé* **Matterhorn**<br>• Microbes for healthy soils and healthy guts John Crawford *Rothamsted,* Joachim Lammel *Yara,* Doug Cook *UC Davis,* Maurice Moloney *GIFS* **Lavaux**<br>• Water conservation and stewardship for agriculture Wouter Wolters, *University Wageningen*, Adrian Sym *AWS*, Frank Eyhorn, *Helvetas,* James Lomax *UN Environment* **Plenary area**<br>• Voices of our Farmers Robert Craig, Ambrose Kirobi CMS, Peter Froehlich *Agricircle,* Martin Mwangi *Wageningen University* **Leman** |
| 12:30-13:30 | *Lunch* | |
| 13:30-14:15 Link to the webcast | **Plenary** Plant breeding of the future | Doug Cook *UC Davis*, Maurice Moloney *Global Inst. Food Security* **Plenary Area** |
| 14:15-15:00 Link to the webcast | **Plenary** Agripreneurship | Evelyn Ohanwusi, Frederick Schreurs *IITA* **Plenary Area** |
| 15:00-15:30 | *Refreshment break* | |
| 15:30-17:00 | **Deep Dive Session 2**<br>**From Mechanisation & Chemistry to Biology & Technology**<br>Choice of 4 workshops | • Organic. Costing the Earth Hans Herren, *Biovison,* Samuel Vionnet, *Valuing Nature,* Urs Niggli, *FIBL,* Vanja Westerberg, Altus Impact **Plenary Area**<br>• Modelling of the food system *Joost Vervoort, Uni. Oxford & Utrecht,* Gerald Nelson *IFPRI,* Natalia Brzezina, *KU Leuven,* Karen Cooper, *Nestlé* **Leman**<br>• Productivity, transparency and proximity through technology, Aileen Ionesco-Somers, *Business School Lausanne*, Michiel Bakker *Google,* Sara Roversi, *Future Food institute,* Rob Skidmore, *ITC,I* Robert Erhard, *Nestlé* **Matterhorn**<br>• Animal Proteins, Plant Proteins Ian Roberts *Buhler*, Duncan Williamson *WWF,* Aarti Ramachandran, *FAIRR,* Patrick Holden, *Sustainable Food Trust,* Leonidas Karagounis *Nestlé* **Lavaux** |
| 17:00 | *End of Day 1* | |
| 19:00-22:00 | *Dinner at **nest museum*** (see plan p4) | Speaker: Jeremy Oppenheim Systemiq |

| Day 2: July 7 | Theme Day 2: Healthy & Sustainable Diets | |
|---|---|---|
| 08:00-08:30 | *Coffee, refreshments* | |
| 08:30-8.45 Link to the webcast | Welcome and opening remarks | Heiko Schipper, *Head of Nestlé Nutrition* **Plenary Area** |
| 08:45-10.15 Link to the webcast | **Plenary: Setting the Scene II** Food system or Nutrition system?<br><br>Nutrition at the Nexus<br><br>Nutritional quality of crops | Heiko Schipper **Plenary Area**<br><br>Jessica Fanzo *John Hopkins University*<br><br>Bev Postma, *HarvestPlus* |
| 10:15- 10:45 | *Refreshment break* | |
| 10:45-12:15 | **Deep Dive Session 3 More Food in Food**<br>Choice of 4 workshops | • Urban and rural nutrition challenges Frank Mechielsen *Hivos*, Adam Drewnowski, *University of Washington,* Hernan Manson *ITC,* Wilbert Sybesma, *Nestlé* **Matterhorn**<br>• Responsible sourcing Rob Cameron *SustainAbility,* Joost Oorthuizen, *IDH,* Christina Nyhus Dhillon*, GAIN,* Benjamin Ware, Nancy Madigu, *Nestlé* **Plenary Area**<br>• Food loss and food waste Richard Swannell *WRAP,* Franziska Staubli,*Migros,* Clementine O'Connor, Achim Drewes, *Nestlé* ***Lavaux***<br>• Nutrition & Climate Change Josette Lewis, *Env. Defense Fund,* Doug Cook, *UC Davis,* Gerald Nelson *IFPRI,* Pierre Herben *Yara* ***Leman*** |
| 12:15-13:00 | *Lunch* | |
| 13:00-14:00 Link to the webcast | Responsible Investments in Agriculture & Food Systems | **Plenary Area**<br>Francesco Pierri *FAO,* Coralie David, *responsAbility,* Nabeela Khan *CDC,* Ben Valk, *Rabobank* |
| 14:00-16:30 Link to the webcast | **Plenary Panels Processed Food, Personalised Nutrition** | |
| 14:00-14:45 Link to the webcast | Engaging the consumer's brain | Olivier Oullier, *University of Aix Marseille* **Plenary Area** |
| 14:45-15:30 Link to the webcast | Processed food | Adam Drewnowski *Uni. Washington,* Natalie Kindred *HBS*, Wayne England, *SBU Food*, Stefan Palzer, *Nestle Research* **Plenary Area** |
| *15:30-15:45* Link to the webcast | *Refreshment break* | |
| 15:45-16:30 Link to the webcast | Personalised nutrition | Valerio Nannini, *Nestlé Research* **Plenary Area** |
| 16:30-17:00 Link to the webcast | **Closing remarks** | Stefan Catsicas, *CTO Nestlé* **Plenary Area** |

# Event layout

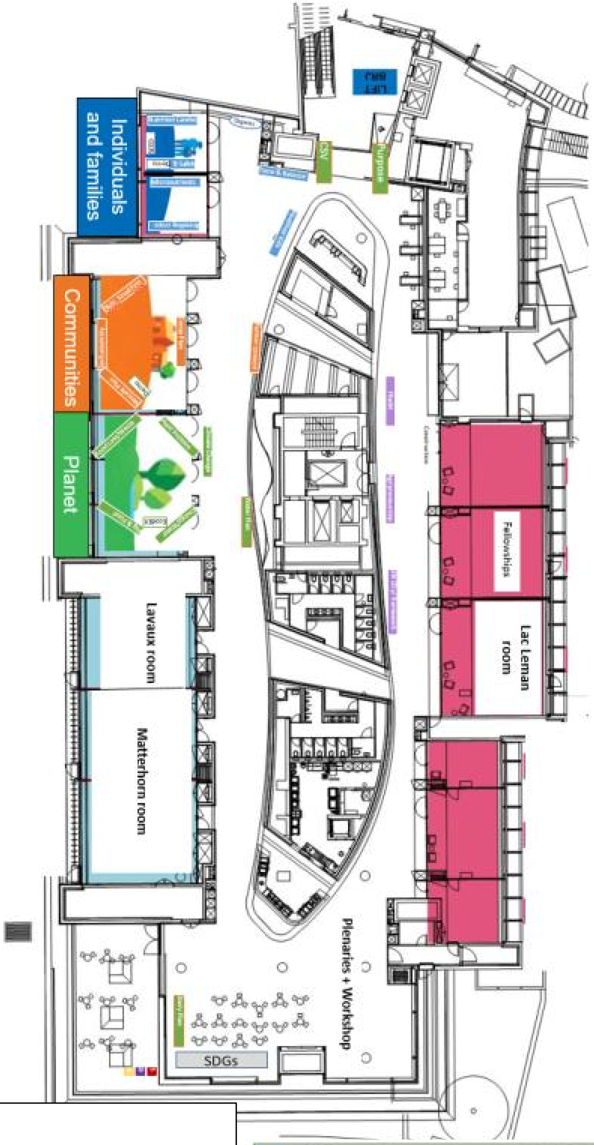


**The nest address** :
Chaussée de la Guinguette 10, 1800 Vevey

nest museum is located right behind Vevey's train station and is accessible by foot by crossing the train station towards platform 7. You will then need less than 2 minutes to reach the museum

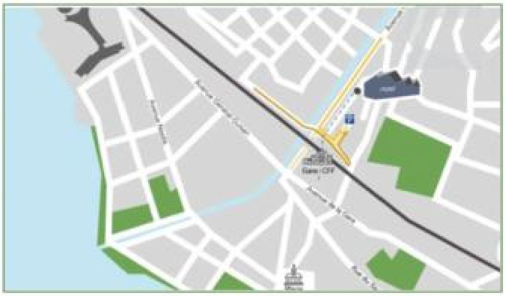

Supplement: Supplementary file 1 — Appendix S1. Supporting information [file JSFA-98-3219-s001.pdf]
